# Healthcare-seeking Behaviour for Common Infectious Disease-related Illnesses in Rural Kenya: A Community-based House-to-house Survey

**DOI:** 10.3329/jhpn.v29i1.7567

**Published:** 2011-02

**Authors:** Deron C. Burton, Brendan Flannery, Bernard Onyango, Charles Larson, Jane Alaii, Xingyou Zhang, Mary J. Hamel, Robert F. Breiman, Daniel R. Feikin

**Affiliations:** ^1^ Centers for Disease Control and Prevention, 1600 Clifton Road NE, Atlanta, GA 30333, USA; ^2^ Kenya Medical Research Institute/CDC Research and Public Health Collaboration, Kisumu, Kenya; ^3^ McGill Medical School, Montreal, Canada; ^4^ Kenya Medical Research Institute, Kisumu, Kenya; ^5^ CDC International Emerging Infections Program, Nairobi, Kenya; ^6^ McKing Consulting Corporation, 2900 Chamblee Tucker Road, Building 10, Suite 100, Atlanta, GA 30341, USA

**Keywords:** Community health services, Delivery of healthcare, Diarrhoea, Fever, Healthcare-seeking behaviour, Pneumonia, Rural health, Rural health services, Kenya

## Abstract

Community surveys of healthcare-use determine the proportion of illness episodes not captured by health facility-based surveillance, the methodology used most commonly to estimate the burden of disease in Africa. A cross-sectional survey of households with children aged less than five years was conducted in 35 of 686 census enumeration areas in rural Bondo district, western Kenya. Healthcare sought for acute episodes of diarrhoea or fever in the past two weeks or pneumonia in the past year was evaluated. Factors associa-ted with healthcare-seeking were analyzed by logistic regression accounting for sample design. In total, 6,223 residents of 981 households were interviewed. Of 1,679 children aged less than five years, 233 (14%) had diarrhoea, and 736 (44%) had fever during the past two weeks; care at health facilities was sought for one-third of these episodes. Pneumonia in the past year was reported for 64 (4%) children aged less than five years; 88% sought healthcare at any health facility and 48% at hospitals. Seeking healthcare at health facilities was more likely for children from households with higher socioeconomic status and with more symptoms of severe illness. Health facility and hospital-based surveillance would underestimate the burden of disease substantially in rural western Kenya. Seeking healthcare at health facilities and hospitals varied by syndrome, severity of illness, and characteristics of the patient.

## INTRODUCTION

Nearly 10 million children in developing countries die each year before reaching five years of age, mostly from pneumonia, diarrhoea, and malaria (1, 2). Implementation of proven interventions would reduce mortality due to these common childhood illnesses (3–7). However, the potential impact of such interventions in different locations in Africa is difficult to estimate without accurate local disease-burden data. Obtaining such estimates is challenging in much of Africa where ill children often never access formal healthcare, and most deaths occur at home (8, 9). Accurate estimates of the burden of diseases are needed to set public-health priorities for allocation of resources.

Health facility-based surveillance of diseases is the most common method for assessing the burden of diseases. Facility-based surveillance allows verification of signs and symptoms by trained clinicians and simplifies collection of specimens to confirm diagnoses. Despite these advantages, facility-based surveillance underestimates the actual burden of diseases in places where healthcare-use is poor. Results of healthcare-use surveys can be used for adjusting facility-based measurements of the burden of diseases (10–12). Healthcare-use surveys can also inform policy-makers about the potential impact of facility-based interventions, such as Integrated Management of Childhood Illness (IMCI) (13, 14).

To guide local decisions about the type of surveillance to be conducted for infectious disease syndromes in a rural district in western Kenya, we carried out a community survey of healthcare-use for acute episodes of fever, respiratory illness, and diarrhoea among young children and their household members. We estimated the proportion of acute episodes likely to be captured by a health facility- and hospital-based surveillance.

## MATERIALS AND METHODS

### Study setting

The survey was conducted in Bondo district in rural western Kenya. The district has borders with Lake Victoria and includes several islands. The total popu-lation of the district was approximately 239,000 in 1999 (15). The population is predominantly of Luo ethnicity. Subsistence farming and fishing are the main economic activities. The prevalence of HIV is among the highest in Kenya (11% in men aged 13-34 years and 21% in women aged 13-34 years in 2003) (16), and malaria is hyperendemic (17). In 2005, the rate of mortality of children aged less than five years from a demographic surveillance system in part of Bondo district was 227 per 1,000 livebirths (18). The district has eight inpatient facilities and 45 outpatient facilities.

### Study design

In August 2005, a sample of households with at least one child aged less than five years (under-five child) was obtained by cluster sampling. The 49 sublocations, comprising 686 enumeration areas within Bondo district (the primary sampling units), were ordered geographically, and 31 sublocations were selected with replacement by probability proportional to estimated total population. In total, 35 enumeration areas were randomly chosen from the 31 sublocations included. Enumeration area-boundaries were defined by the Kenyan Bureau of Statistics for the 1999 national census (19). The number of households per enumeration area ranged from 39 to 199 (median 84).

Trained interviewers visited all households within the selected enumeration areas to identify households with under-five children. Households in which an under-five child resided but for which the child's primary caretaker was not present were revisited once and were treated as non-respondent households if a caretaker was not present on revisit. All the households with an under-five child and a primary caretaker present were included in the sample for all selected enumeration areas. For the enrolled households, interviewers administered questionnaire**s** in the local language (*dho-Luo*) to collect demographic and socioeconomic information, including the educational level of caretakers, primary source of income, access to potable water, and household possessions.

Adult caretakers were asked to identify episodes of acute diarrhoea or fever within the preceding two weeks for all household members. Diarrhoea was defined as three or more loose stools within a 24-hour period. Fever was subjective as perceived by the caretakers. Caretakers were also asked to identify episodes of acute respiratory illness within the preceding 12 months characterized by cough and difficult breathing for more than two days or a diagnosis of ‘pneumonia’ by a healthcare worker. The respiratory screening questions were based on the definition of probable pneumonia episodes from a validation study of verbal autopsies that included children who died of pneumonia (20). We felt that this definition based on verbal autopsies was likely to capture severe respiratory illness, more likely to be pneumonia. Since we focused on pneumonia, which is relatively rare compared to fever and diarrhoea, we asked about acute respiratory illness in the past 12 months, rather than two weeks. We found that the large majority (71%) of reported respiratory illness episodes occurred less than two weeks before the interview. We also found that the severity of acute respiratory illness was greater for more distant episodes compared to episodes that occurred during the past two weeks (see Results section). Because of these findings, we assumed respiratory illness episodes that occurred more than two weeks in the past were more likely to be pneumonia whereas episodes in the past two weeks were more likely to capture milder respiratory illnesses. Therefore, for analysis, we classified episodes that occurred in the past two weeks as acute respiratory illness (ARI) and episodes in the past 12 months (excluding the past two weeks) as pneumonia.

For each household member with a reported disease episode, caretakers were asked detailed questions about symptoms and healthcare sought, including sources of care, diagnostics performed, treatment received, and whether the household member was hospitalized. Reported visits to healthcare facilities for illness episodes were not validated by cross-checking with facility registers.

### Sample-size

The sample-size was calculated to provide a precision of at least ±0.20 for the proportion of under-five children who sought care at a health facility for an episode of pneumonia in the past 12 months (which was expected to be the least common of the clinical syndromes studied). The survey was designed to include visits to approximately 2,900 households based on the assumptions that 50% of such children would have sought medical care, that 5% of children would have had an episode of pneumonia in the past 12 months, and that 33% of households would have an under-five child. Healthcare-seeking behaviour for each syndrome was assumed to be independent. For children with more than one reported illness episode of the same syndrome, respondents were asked about healthcare sought for the most recent episode. A design effect of 2 was assumed, thus doubling the estimated number of children to be surveyed.

### Statistical analysis

Analysis of data was performed using the SAS software (version 9.1) (SAS Institute Inc., Cary, NC, USA) and SUDAAN release 9.0 (version 7.5) (Research Triangle Institute, NC, USA) to account for clustered sample design and correlations between individuals from the same household. Self-weighting was used based on the probability proportional to size (PPS) sampling scheme. The χ^2^ test was used for differences in proportions. Multivariate logistic regression was used for identifying the factors independently associated with healthcare-seeking behaviours. Dichotomous independent variables included in each multivariate model were: gender, age (<1 year vs 1-4 years for models pertaining to under-five children), household socioeconomic status (defined according to the number of household goods owned), educational level of caretakers, and severity of illness (defined according to the number of symptoms of severe illness reported) (detailed definitions are in Table 4).

### Ethical approval

The ethical review committees of the Kenya Medical Research Institute (KEMRI) and the U.S. Centers for Disease Control and Prevention (CDC) approved the study.

## RESULTS

Visits were made to 2,810 households (97% of households in selected enumeration areas); a primary caretaker was available for interview in 2,360 (84%) households. Of the 2,360 households, 1,009 (43%) had at least one under-five child. Caretakers of 28 (2.8%) households refused to participate. In total, 1,679 under-five children were included from 981 households with an under-five child, and 4,544 persons aged over five years in these households were also included (Table 1).

**Table 1.** Distribution of reported illness syndromes* among healthcare-use survey respondents, by age-category, Bondo district, Kenya, 2005

Age-category (years)†	No. of survey respondents	Illness in the past 12 months$	Illness in the past 2 weeks		
		% with reported pneumonia (95% CI)	% with reported acute respiratory illness (95% CI)	% with reported diarrhoea (95% CI)	% with reported fever (95% CI)
0-4	1,679	3.8 (3.3-4.4)	9.4 (8.2-10.7)	13.9 (12.3-15.7)	43.8 (41.4-46.3)
5-17	2,035	0.9 (0.8-1.1)	5.3 (4.4-6.4)	2.7 (2.1-3.5)	18.0 (16.2-20.0)
18-49	2,203	1.8 (1.5-2.1)	4.4 (3.7-5.2)	3.9 (3.1-4.8)	15.8 (14.2-17.6)
≥50	306	2.0 (1.4-3.0)	6.1 (4.1-8.9)	5.2 (3.2-8.4)	20.6 (16.3-25.6)
All ages	6,223	2.1 (1.9-2.3)	6.1 (5.5-6.8)	6.3 (5.6-7.0)	24.4 (23.1-25.7)

*See Materials and Methods section for definitions of illness syndromes;

†Respondents in all age-categories were from households with at least one under-five child;

$Excludes the past two weeks; CI=Confidence interval

In the participating households, 85% of primary caretakers were female, and 44% had not completed primary school. Forty-one percent of the households were classified as of higher socioeconomic status; radios (79%), bicycles (63%), and furniture (54%) were the most common possessions. Most (86%) households relied on surface-water for drinking, and 59% used pit-latrines (the remainder had no toilet).

Of the 1,679 under-five children, 14% [95% confidence interval (CI) 12-16] had diarrhoea, and 44% (95% CI 41-46) had subjective fever in the past two weeks (Table 1). The median duration of episodes of diarrhoea was three days, and the median duration of fever was four days. Thirty-one percent (n=72) of children with diarrhoea and 26% (n=191) of children with fever met our criteria for severe illness episodes.

Episodes of respiratory illness were reported for 222 (13%) of the 1,679 under-five children, the majority (n=158; 71%) of whom had illness onset**—**cate-gorized as ARI**—**in the two weeks preceding the interview. Compared to ARI episodes, respiratory illness episodes that occurred more than two weeks in the past—categorized as pneumonia—were significantly more likely to have included wheezing (94% for pneumonia vs 79% for ARI; p=0.01), to have met our criteria for severe respiratory illness (40% vs 20%; p=0.01), to have been hospitalized (26% vs 7%; p< 0.01), and to have been diagnosed with pneumonia by a healthcare worker (86% vs 18%; p<0.01). ARI episodes also tended to have lower mortality (2%) than pneumonia episodes (8%; p=0.06).

All illness syndromes were reported less frequently among household members aged five years or older compared to under-five children (Table 1).

Of the under-five children, complete data on healthcare-seeking behaviour were available for 78% of respiratory illness episodes, 81% of diarrhoeal episodes, and 81% of febrile episodes (syndromes not mutually exclusive). Treatment at a health facility was sought in 35-42% of illnesses that occurred during the preceding two weeks (Table 2). Care was slightly more commonly sought at hospitals for ARI (18%, 95% CI 12-26) than for diarrhoea (8%, 95% CI 5-13) or fever (11%, 95% CI 9-14). Common sources of healthcare-seeking outside the household for ARI, diarrhoea, or fever were drug-sellers (range 39-45%) and private care providers (range 22-24%). A relatively few children visited traditional healers (range 3-7%) or village health volunteers (range 5-6%) (Table 2).

**Table 2.** Healthcare-seeking behaviours of caretakers of under-five children, by reported illness, Bondo district, Kenya, 2005

Healthcare-seeking behaviour	Past 12 months*	Past 2 weeks		
	% of children with reported pneumonia (95% CI) (n=50)	% of children with reported acute respiratory illness (95% CI) (n=123)	% of children with reported diarrhoea (95% CI) (n=188)	% of children with reported fever (95% CI) (n=589)
Healthcare-seeking behaviours outside household				
Any care sought outside household	100	85.4 (77.1-91.0)	85.7 (79.8-90.1)	85.4 (82.3-88.0)
Any health facility visitation	88.0 (75.7-94.5)	41.5 (32.6-50.9)	36.0 (29.3-43.2)	35.2 (31.4-39.3)
Hospital visitation	48.0 (34.8-61.5)	17.9 (11.9-25.9)	7.9 (4.9-12.7)	11.2 (8.9-14.1)
Hospitalization	26.0 (15.9-39.6)	6.5 (3.3-12.5)	1.6 (0.5-4.8)	3.4 (2.1-5.2)
Private care providers	24.0 (14.2-37.5)	22.8 (15.8-31.7)	24.3 (18.6-31.2)	22.1 (18.8-26.0)
Drug-sellers	20.0 (11.3-33.0)	39.0 (30.3-48.6)	45.0 (37.9-52.3)	45.0 (40.8-49.2)
Traditional healers	4.0 (1.0-14.8)	3.3 (1.2-8.4)	7.4 (4.3-12.5)	6.9 (5.0-9.4)
Village health volunteers	2.0 (0.3-13.0)	4.9 (2.2-10.5)	6.3 (3.7-10.7)	6.0 (4.3-8.3)
Reported diagnostics				
Chest radiograph	6.0 (1.9-17.1)	3.3 (1.2-8.2)	0	0.7 (0.3-1.8)
Blood smear	0	17.9 (11.9-26.0)	15.3 (10.7-21.6)	16.6 (13.8-19.9)
Reported treatments				
Antibiotics	44.0 (30.8-58.1)	61.0 (51.8-69.4)	62.4 (55.0-69.4)	56.0 (51.8-60.2)
Antimalarial medication	66.0 (51.7-77.9)	59.3 (50.0-68.1)	52.4 (45.2-59.5)	60.9 (56.8-64.9)
Oral rehydration therapy	20.0 (11.1-33.4)	17.9 (11.6-26.5)	32.3 (25.9-39.4)	12.8 (10.2-15.8)
Intravenous fluid	0	2.4 (0.8-7.3)	2.6 (1.1-6.2)	1.0 (0.5-2.2)
Herbal/traditional remedies	40.0 (27.3-54.2)	34.1 (25.6-43.8)	40 (33-48)	35.1 (31.1-39.4)
Prayer	40.0 (27.3-54.2)	26.0 (18.5-35.2)	32.8 (26.1-40.3)	27.9 (24.1-32.0)

*Excludes two weeks before interview

For pneumonia episodes in the preceding 12 months (excluding ARI in the preceding two weeks), the caretakers reported seeking healthcare outside the home for all episodes. Significantly more of these episodes resulted in visits to health facilities (88%, 95% CI 76-95), visits to hospitals (48%, 95% CI 35-62), and hospitalizations (26%, 95% CI 16-40) than for ARI in the past two weeks (Table 2). Of the 13 children with reported pneumonia who were hospitalized, nine (69%) had first visited an outpatient health centre for care.

Diagnostic procedures were uncommonly reported for all syndromes. Of children with febrile illnesses, blood smears were reported for 17% of episodes. Chest radiographs for pneumonia and tests of stool specimens for diarrhoea episodes were performed in 6% and 2% of episodes respectively (Table 2). In contrast, intake of antibiotics and antimalarials was common. Of children with ARI, diarrhoea, or fever in the past two weeks, 61%, 62%, and 56% took an antibiotic and 59%, 52%, and 61% took an antimalarial drug respectively. Children with any of the reported illnesses often received herbal reme-dies (range 34-40%) or were prayed for with regard to their illness (range 26-40%) (Table **2**).

Compared to under-five children, other household members aged five years or older had generally similar patterns of most healthcare-seeking behaviours (Table 3). However, persons aged over five years visi-ted health facilities less often than young children for episodes of pneumonia (66% vs 88%, p=0.01).

**Table 3.** Healthcare-seeking behaviours of persons aged 5 years or older*, by reported illness, Bondo district, Kenya, 2005

Healthcare-seeking behaviour	Past 12 months†	Past 2 weeks		
	% of persons with reported pneumonia (95% CI) (n=50)	% of persons with reported respiratory illness (95% CI) (n=168)	% of persons with reported diarrhoea (95% CI) (n=115)	% of persons with reported fever (95% CI) (n=603)
Healthcare-seeking behaviours outside household				
Any care sought outside household	92.0 (81.2-96.8)	84.6 (77.9-89.6)	76.5 (67.0-84.0)	80.6 (76.4-84.3)
Any health facility visitation	66.0 (52.8-77.1)	44.4 (35.9-53.2)	33.0 (24.3-43.1)	35.9 (31.4-40.7)
Hospital visitation	34.0 (22.5-47.7)	13.0 (8.5-19.5)	4.3 (1.8-10.0)	9.6 (7.2-12.7)
Hospitalization	16.0 (8.3-28.7)	5.3 (2.6-10.5)	4.3 (1.8-10.0)	4.0 (2.6-6.0)
Private care providers	32.0 (20.6-46.1)	16.0 (11.2-22.3)	10.4 (6.0-17.5)	17.5 (14.5-21.1)
Drug-sellers	36.0 (23.7-50.5)	42.0 (34.2-50.3)	36.5 (27.8-46.3)	42.7 (38.1-47.5)
Traditional healers	10.0 (4.2-22.0)	5.9 (3.2-10.6)	4.3 (1.2-14.5)	4.0 (2.5-6.4)
Village health volunteers	4.0 (1.0-14.7)	3.0 (1.2-6.9)	6.1 (2.9-12.3)	5.1 (3.6-7.3)
Reported diagnostics				
Chest radiograph	14.0 (7.0-26.1)	5.9 (3.2-10.6)	1.7 (0.4-6.6)	1.7 (0.8-3.2)
Blood smear	6.0 (1.9-17.1)	13.0 (8.6-19.3)	9.6 (5.2-17.0)	14.6 (11.7-18.0)
Reported treatments				
Antibiotics	58.0 (43.8-71.0)	52.1 (44.3-59.8)	58.3 (49.1-66.9)	50.7 (46.1-55.3)
Antimalarial medication	78.0 (64.2-87.5)	57.7 (49.3-65.1)	53.0 (43.4-62.5)	68.4 (63.9-72.5)
Oral rehydration therapy	10.0 (4.3-21.7)	5.9 (3.2-10.6)	21.7 (15.0-30.4)	6.1 (4.4-8.5)
Intravenous fluid	0	0.6 (0.1-4.1)	7.8 (4.1-14.4)	1.0 (0.4-2.2)
Herbal/traditional				
remedies	42.0 (29.1-56.1)	30.2 (22.8-38.8)	36 (27-45)	24.0 (20.3-28.2)
Prayer	26.0 (16.0-39.4)	35.5 (27.7-44.2)	20.0 (13.2-29.1)	25.2 (21.0-29.8)

*Only members of households with under-five children were included (see Materials and Methods section);

†Excludes two weeks before interview

Table 4 shows the proportions of under-five res-pondents with syndromes of interest who visited health facilities, stratified by gender, age, socioeconomic status, educational level of caretakers, and severity of illness. In multivariate analyses, including all these factors simultaneously, household socioeconomic status and severity of illness were independently associated with seeking healthcare at health facilities (significant independent associations are denoted by single asterisks in Table 4). For pneumonia episodes, children in households with higher socioeconomic status were more likely to be taken to any health facility [adjusted odds ratio (AOR)=21.8, 95% CI 1.0-456.9] or to be taken to a hospital (AOR=4.1, 95% CI 1.4-12.2) and be hospitalized (AOR=7.3, 95% CI 1.1-48.1). Children with more severe pneumonia were more likely to be taken to a hospital than children with less severe pneumonia (AOR=5.9, 95% CI 1.4-25.2). For diarrhoea episodes, higher socioeconomic status was associated with seeking healthcare at a hospital (AOR=3.4, 95% CI 1.0-10.8). For febrile episodes, more severe illness was associated with more hospitalizations (AOR=3.7, 95% CI 1.4-9.4). Healthcare was more often sought for male children, although the differences were not significant (Table 4). Of other household members aged five years or older, households with higher socioeconomic status were associated with a higher probability of visiting any health facility for febrile illness; greater severity of illness was associated with a higher probability of visiting any health facility for febrile illness and a higher probability of visiting a hospital for pneumonia (data not shown).

**Table 4.** Factors associated with healthcare-seeking behaviours for children aged less than 5 years, by reported illness, Bondo district, Kenya, 2005

Characteristics	Past 12 months†			Past 2 weeks								
	Reported pneumonia (n=50)			Reported respiratory illness (n=123)			Reported diarrhoea (n=188)			Reported fever (n=589)		
	% visiting any facility	% visiting hospital as in- or outpatient	% hospitalized	% visiting any facility	% visiting hospital as in- or outpatient	% hospitalized	% visiting any facility	% visiting hospital as in- or outpatient	% hospitalized	% visiting any facility	% visiting hospital as in- or outpatient	% hospitalized
All under-five												
children	88.0	48.0	26.0	41.5	17.9	6.5	36.0	7.9	1.6	35.2	11.2	3.4
Gender												
Male	95.0	55.0	30.0	39.3	19.7	9.8	38.2	12.4	3.4	34.7	13.9	4.5
Female	83.3	43.3**	23.3	43.5	16.1	3.2	34.3	4.0**	0	35.9	8.8**	2.3
Infant aged less than 1 year
Yes	87.5	50.0	25.0	42.9	28.6	14.3	36.6	4.9	0	43.4	12.1	4.0
No	88.1	47.6	26.2	41.2	15.7	4.9**	35.8	8.8	2.0	33.6**	11.1	3.2
Socioeconomic status$												
High	96.4	53.6	32.1	48.0	28.0	12.0	36.7	13.9	2.5	36.5	14.6	3.9
Low	77.3*	40.9	18.2	37.0	11.0*	2.7*	35.5	3.6*	0.9	34.4	9.1**	3.0
Education of caretaker‡												
High	83.3	53.3	30.0	37.1	17.1	5.7	37.7	11.3	2.8	36.9	12.7	3.6
Low	95.0**	40.0	20.0	47.2	18.9	7.5	33.7	3.6	0	33.2	9.5	3.1
Severity of illness^$$^												
Severe	90.0	65.0	35.0	36.0	12.0	4.0	35.6	11.9	5.1	38.7	15.5	7.1
Nonsevere	86.7	36.7*	20.0	42.9	19.4	7.1	36.2	6.2	0	34.0	9.8**	2.0*

* p<0.05 in multivariate model, including all five characteristics in the left column;

** p<0.1 in multivariate analysis;

† Excludes two weeks before interview;

$Households of higher socioeconomic status were defined as those with three or more of the following possessions: telephone, television, radio, sofa, bicycle, motorcycle, or car;

‡Caretaker's education was stratified as completion of primary school or higher versus no education or incomplete primary school education;

$$Illness episodes were stratified into severe and non-severe categories according to the number of symptoms of severe illness (adapted from IMCI guidelines) reported by the caretakers (30,31). For under-five children, respiratory illness episodes were defined as severe if they included six or more of the following symptoms: rapid breathing, blue mouth or fingers, inability to breastfeed or drink, vomiting, lethargy, convulsions, loss of consciousness, and chest-pain (for under-two children, grunting, lower chest wall-indrawing, or nasal flaring was substituted for chest-pain). Severe diarrhoeal illness episodes included five or more of the following: blood in stool, sunken eyes (as a sign of dehydration), restlessness or irritability, inability to breastfeed or drink, lethargy, and loss of consciousness. Severe febrile illness episodes included four or more of the following: bleeding from any orifice, cough, difficulty breathing, rapid breathing, vomiting, restlessness or irritability, convulsions, and loss of consciousness. For children and adults aged five years or above (data not shown in table), the minimum numbers of symptoms used in defining severe illness were 4, 4, and 3 for respiratory illnesses, diarrhoeal illness, and febrile illness respectively.

IMCI=Integrated management of childhood illness

## DISCUSSION

This study of healthcare-seeking behaviours in rural Kenya demonstrated that the proportions of illness episodes captured through health facility-based surveillance would vary for different clinical syndromes and surveillance settings. Based on our survey data, surveillance encompassing all healthcare facilities (including outpatient clinics) in Bondo district would capture roughly 30-40% of episodes of diarrhoea, febrile illness, and ARI in under-five children and a larger proportion (approaching 90%) of pneumonia episodes. However, surveillance restricted to inpatient hospitals would capture a much smaller proportion of episodes, from roughly 50% of children with pneumonia to 10-20% of children with less severe respiratory illness, diarrhoea, or malaria. As expected, adults included in the survey less commonly sought care at facilities than children, even for episodes classified as severe.

The prevalence of acute illnesses and patterns of health facility-use for under-five children in this study were similar to other data from Kenya. In the 2003 Demographic and Health Survey, rates of prevalence for acute illness episodes among young children in the past two weeks were 18% for ARI, 41% for fever, and 16% for diarrhoea (19). Treatment was sought at a healthcare facility for 46% of those with ARI and 30% of those with diarrhoea, of whom 29% received oral rehydration salts. In another study in Kakamega district in rural western Kenya, treatment was sought in health facilities for 27% of preschool children with cough or ‘cold’ in the past week, 33% of those with fever, and 40% of those with acute diarrhoea (21). Children may not be taken to hospitals for severe illness episodes for various reasons. Caregivers may not recognize signs and symptoms of severe illness, hospitals may be very far or very costly, or healthcare workers may not refer sick children (22). Higher levels (87%) of hospital-use were found in a rural province in Thailand among children aged less than 14 years with self-reported pneumonia in the past year (10). In middle-income countries, such as Thailand, with good access to hospitals even in rural communities, adjustment for local healthcare-seeking behaviours are likely to result in smaller increases in facility-based estimates of pneumonia incidence than in rural African settings.

A similar, contemporaneous community survey of healthcare-use among children was conducted in a crowded informal settlement in Nairobi (23). The use of healthcare facilities was higher in the urban site than that in the rural site for recent episodes of diarrhoea and of fever among under-five children. The greater use of healthcare in the urban area might be related to the shorter distances needed to be travelled to visit a health facility or a higher level of education, socioeconomic status, or health awareness among the urban population (24). In contrast, frequency and sites of healthcare-seeking for pneumonia and ARI among under-five children were similar in the urban and rural sites in Kenya, with the exception of the fact that children in Bondo district were more often taken to private care providers. Why healthcare-seeking was not more common in the urban site for respiratory illness as it was for fever and diarrhoea is not clear but could be related to differences in the definitions of pneumonia and ARI used in the two studies.

Our study demonstrated that the poorest segments of the community, who are likely at the highest risk of disease, are likely to be under-represented in facility-based surveillance data. Other studies in both rural and urban areas of sub-Saharan Africa have documented similar associations between socioeconomic indicators and healthcare-seekingbehaviours for young children, even in environments that might appear uniformly impoverished (13, 21, 25). We also observed trends towards increased hospital-use for boys compared to girls; although not statistically significant, this is consistent with trends noted in other poor communities in Kenya (25). Although distance to health facilities was not evaluated in this survey, distance was an important determinant of healthcare-seeking behaviour for pneumonia and other syndromes in other studies (26–28). Understanding the determinants of healthcare-seeking at health facilities is necessary to interpret results of facility-based surveillance as those who access facilities might not be representative of the general population.

### Limitations

This study had several limitations. Syndromes were defined based on reported symptoms and were not verified by medical examinations. For example, in malaria-endemic areas, signs and symptoms of pneumonia and malaria in children overlap (20, 29). Second, caretakers’ recall of specific symptoms and healthcare-seeking behaviours may be unreliable, particularly with regard to the reported pneumonia episodes that occurred up to 12 months before the survey. Third, the sample of older children and adults included in the survey was restricted to those living with under-five children and may differ in the burden of disease and healthcare-seeking practices from older children and adults in households without under-five children. Fourth, the socioeconomic status metric used in our multivariate analyses was limited to a single dimension of wealth (household goods owned) and was not externally validated. Last, because of small numbers of respondents in some categories, the precision of AORs from our multivariate analyses was low. Nevertheless, we observed significant independent associations that highlight sub-populations at an increased risk of being missed by facility-based surveillance for the syndromes of interest. Additional research is needed to more closely examine the magnitudes of these associations.

### Conclusions

We found that facility-based surveillance, particularly using hospitals as the catchment sites, would underestimate the true burden of infectious diseases in settings like Bondo district in rural western Kenya. Moreover, facility-based surveillance will differentially underestimate the burden of diseases, based on characteristics of the disease (e.g. severity) and the person (e.g. socioeconomic status and sex). Data from community healthcare-use surveys such as ours may be used for guiding public-health surveillance strategies. For instance, based on local healthcare-use data, surveillance programmes that seek to define the burden of diseases or study aetiologies might consider capturing illness episodes outside facilities, such as at drug-shops and homes, to better estimate the full spectrum of a disease. Moreover, if facility-based surveillance is conducted, community healthcare-use surveys can improve interpretation of surveillance data by providing an adjustment factor that accounts for the proportion of illness episodes for which care is not sought at health facilities. In this way, local healthcare-use data may play an important role to better inform policy-makers on the true burden of diseases to be used in the planning of interventions and evaluation activities.

## ACKNOWLEDGEMENTS

This paper is approved with the permission of the Director, KEMRI.
